# Default mode network alterations after intermittent theta burst stimulation in healthy subjects

**DOI:** 10.1038/s41398-020-0754-5

**Published:** 2020-02-24

**Authors:** Aditya Singh, Tracy Erwin-Grabner, Grant Sutcliffe, Walter Paulus, Peter Dechent, Andrea Antal, Roberto Goya-Maldonado

**Affiliations:** 1 Laboratory of Systems Neuroscience and Imaging in Psychiatry, Department of Psychiatry and Psychotherapy of the University Medical Center Göttingen, Göttingen, Germany; 2 Department of Clinical Neurophysiology of the University Medical Center Göttingenn, Göttingen, Germany; 3 Core facility ‘MR-Research in Neurology and Psychiatry’, Department of Cognitive Neurology of the University Medical Center Göttingenn, Göttingen, Germany

**Keywords:** Neuroscience, Depression

## Abstract

Understanding the mechanisms by which intermittent theta burst stimulation (iTBS) protocols exert changes in the default-mode network (DMN) is paramount to develop therapeutically more effective approaches in the future. While a full session (3000 pulses) of 10 Hz repetitive transcranial magnetic stimulation (HF-rTMS) reduces the functional connectivity (FC) of the DMN and the subgenual anterior cingulate cortex, the current understanding of the effects of a single session of iTBS on the DMN in healthy subjects is limited. Here, we use a previously validated target selection approach for an unprecedented investigation into the effects of a single session (1800 pulses) of iTBS over the DMN in healthy controls. Twenty-six healthy subjects participated in a double-blind, crossover, sham-controlled study. After iTBS to the personalized left dorsolateral prefrontal cortex (DLPFC) targets, we investigated the time lapse of effects in the DMN and its relationship to the harm avoidance (HA) personality trait measure (Temperament and Character Inventory/TCI). Approximately 25–30 min after stimulation, we observed reduced FC between the DMN and the rostral and dorsal anterior cingulate cortex (dACC). About 45 min after stimulation the FC of rostral and dACC strongly decreased further, as did the FC of right anterior insula (AI) with the DMN. Also, we report a positive correlation between the FC decrease in the rostral ACC and the HA domain of TCI, indicating that the HA scores can potentially predict iTBS response. Overall, our results show the time lapse by which iTBS at left-DLPFC targets reduces the FC between DMN and the dACC and right AI, regions typically described as nodes of the salience network.

## Introduction

The large variability of responses to the FDA-approved 10 Hz repetitive transcranial magnetic stimulation (rTMS) protocol for the treatment of depression has led to a world-wide demand for better techniques or improved protocols. The non-inferior antidepressant efficacy of the 3 min/session theta burst protocol^1^ compared to 37.5 min/sessions of conventional 10 Hz rTMS protocol has played a role in increasing the use of the theta burst protocol for antidepressant treatment^2–5^. Nevertheless, brain connectivity changes underlying the effects of intermittent theta burst stimulation (iTBS) delivered at the left dorsolateral prefrontal cortex (DLPFC) remain unexplored. Many factors contribute to inter-individual variability, including natural variation in anatomy and functional connectivity. Here we use a previously validated target selection method to improve precision of coil localization and investigated the effects of iTBS on the relevant brain networks that best cover the left DLPFC and the anterior cingulate cortex (ACC).

The TBS protocol was developed to mimic rodent^6,7^ and human hippocampal activity^8^, where a combination of gamma-frequency spike patterns superimposed on theta rhythms^9^ was found. It involves application of a burst of three TMS pulses every 20 ms (50 Hz), which is repeated five times per second (5 Hz)^10,11^. When delivered continuously (continuous TBS–cTBS) for 40 s, it results in reduced corticospinal excitability, while when administered in an intermittent fashion (iTBS) it results in increased corticospinal excitability^9^. Studies of TBS stimulation on motor cortex have shown plasticity changes beyond the duration of stimulation typically lasting in the range of 30 min^11,12^.

Beyond local effects under the stimulation coil, plasticity changes in brain’s altered functional connectivity away from the stimulation point, e.g. the DLPFC^13^, are likely relevant to the treatment of depression, which has been associated with aberrant brain functional connectivity^14^. The default-mode network (DMN), consisting of the medial prefrontal cortex, posterior cingulate cortex, and areas of posterior parietal cortex^15^. Using 10 Hz rTMS as antidepressant treatment, a study has recently replicated the prediction of symptomatic alleviation in depression when aberrant sgACC connectivity with the DMN is decreased, which happened in responders but not in non-responders^18^. Furthermore, such effects over networks in healthy subjects have been shown in our previous work using 10 Hz rTMS^19^. Already after a single session of 10 Hz rTMS (3000 pulses), significant reduction in the connectivity between the sgACC and the DMN was evidenced.

Given the central involvement of the DMN in the pathophysiology of depression and the importance of a shorter protocol such as iTBS for reducing symptoms^15,20–31^, here we aimed to uncover connectivity effects of a single session of a prolonged iTBS protocol (1800 pulses) in healthy subjects. ITBS is therapeutically beneficial for depression^1^, which is characterized by a hyperconnected DMN^15,16^, and treatment for depression is accompanied by normalization of this hyperconnectivity^17^. Assuming the therapeutic mechanism of iTBS shares the same mechanism of action in healthy subjects, we hypothesize that iTBS would act by reducing the functional connectivity of DMN.

We applied a single session of iTBS at left DLPFC sites and analyzed the DMN during three time-windows after stimulation in a double-blind, crossover, and sham-controlled study. To the best of our knowledge time lapse of effects from iTBS at left DLPFC have not been previously reported. We expect that depicting the acute effects after one session of iTBS stimulation in healthy subjects will be crucial to understand the relationship between the stimulation site and the sgACC across time, up to ~50 min after stimulation^32^. Further comprehension of these network dynamics could help to inform and promote more efficient and tailored iTBS interventions in the future. Considering our previous results from a single session of 10 Hz rTMS, we expected to see maximum effects of a single session of iTBS after approximately 30 min. We hence acquired a resting state functional MRI (rsfMRI) at 27–32 min after stimulation. However, on exploratory basis, we also wanted to investigate potential changes during other time windows. Particularly, to document iTBS induced changes before and after the expected 30-min mark, we acquired one rsfMRI from 10 to 15 min (earliest possible time point after stimulation) and another from 45 to 50 min after stimulation.

Lastly, harm avoidance (HA) of the Temperament and Character Inventory (TCI) pertains to the heritable tendency of individuals to respond more harshly to aversive cues, punishment and non-reward^33^. Previous works show a relationship between HA and activity^34–37^ or connectivity^19^ in sgACC in healthy samples. In patients with depression, HA has been shown to be associated with response^38^ and non-response^39^ to treatment. As we employ a single session of clinically relevant iTBS protocol in healthy subjects, we propose that reduction of DMN connectivity after stimulation would have a relationship to HA. Based on our previously reported^19^ negative relationship between 10 Hz rTMS induced changes in sgACC and HA, we hypothesize that a similar negative relationship would exist between iTBS induced changes and HA scores.

## Materials and methods

### Participants

Healthy subjects between the ages of 18–65 were enrolled in the study. We evaluated the subjects with structured clinical interviews and ruled out current or prior neuropsychiatric disorders and contraindications to rTMS and/or MRI. We performed the experiments in agreement with relevant guidelines and regulations^40,41^. The Ethics Committee of the University of Medical Center Göttingen approved the study protocol and subjects provided their informed consent before investigation.

### Study design

**Fig. 1:**
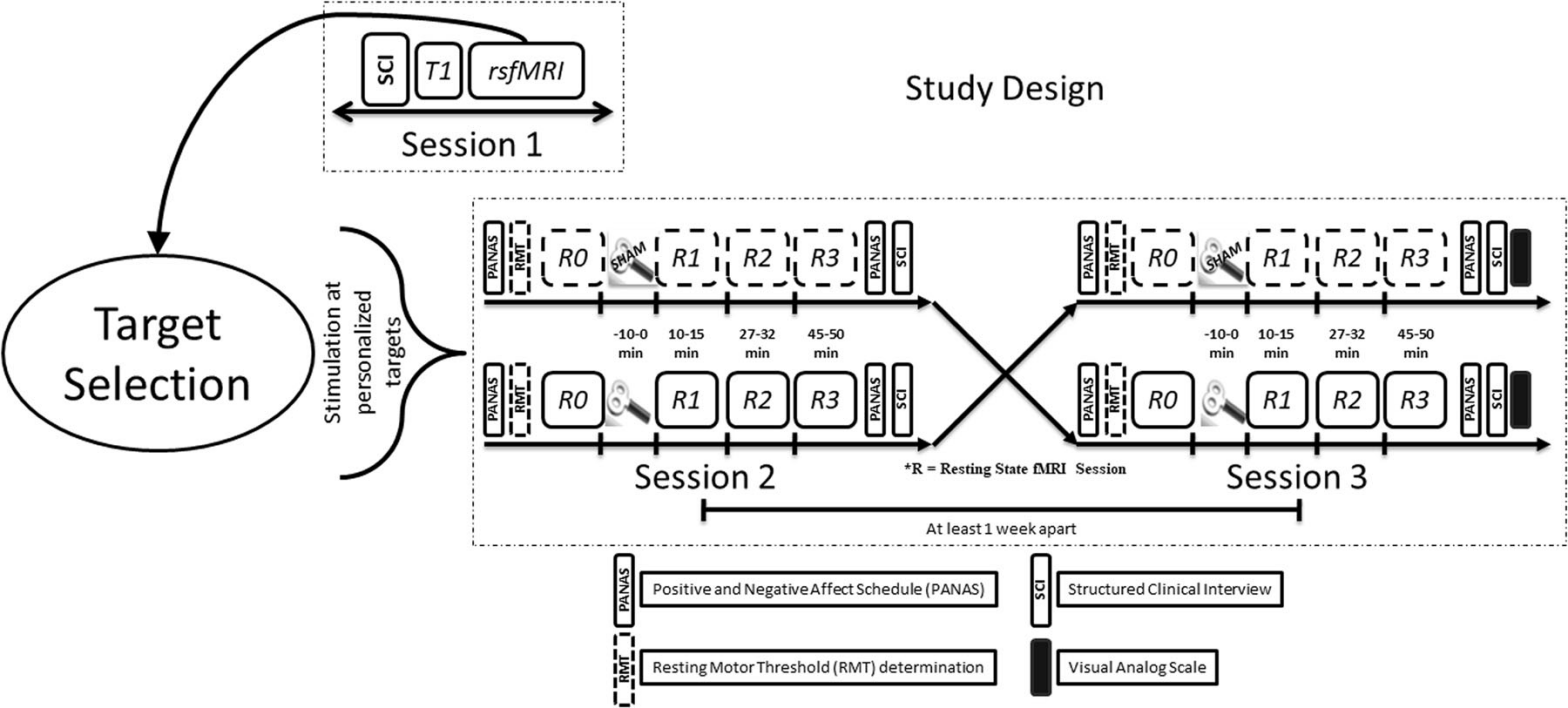
A schematic representation of the study design. In session 1, we obtained the informed consent and collected the information (see main text for details) from Structured Clinical Interviews (SCI). After this we acquired a structural (T1-weighted) and functional (rsfMRI) images. During rsfMRI, the subjects were instructed to fixate at a “+” and mind wander, while their open eyes were monitored by eye tracking. Personalized targets were found using each subject’s rsfMRI as has been described elsewhere^19^. Using online neuronavigation, we delivered real or sham iTBS, in a counterbalanced and pseudo-randomized fashion, at 80% of resting motor threshold. Baseline and three post-iTBS rsfMRI scans were acquired. The subjects also completed the Positive and Negative Affect Schedule (PANAS) both before and after the sessions, and a visual analog scale (VAS) for perceived effects of iTBS on mental state and scalp sensation at the end of the investigation.

The study reported here with healthy subjects is a sham-controlled, double-blind (subject and interviewer were unaware of the stimulation condition), crossover study with real and sham iTBS delivered in a counterbalanced and pseudo-randomized fashion. We conducted the experiments over three sessions (each session on a different day, Fig. 1) with each session separated by at least one week.

### Session 1

In session 1, the interviewer administered a Structured Clinical Interview (SCI) consisting of the Beck Depression Inventory II (BDI II), Montgomery-Asberg Depression Rating Scale (MADRS), Hamilton Depression Rating Scale (HAM-D) and Young Mania Rating Scale (YMRS). In addition to the SCI, to further establish the mental health and well-being of the subjects we asked them to complete the Symptoms Checklist 90-revised (SCL 90-R), Temperament and Character Inventory (TCI), Positive and Negative Syndrome Scale (PANSS), Life Orientation Test – Revised (LOT-R), Barratt Impulsiveness Scale (BIS), a handedness questionnaire^42^ and a vocabulary-based intelligence test (MWT). Next, we acquired structural T1-weighted MRI and resting state functional MRI (rsfMRI) scans for our method of target selection (see Fig. 1 for further details). The process of personalized left DLPFC target selection has been described previously^19^.

### Session 2 and Session 3

To allow washout of any potential iTBS effects, session 2 and session 3 were separated by at least a week. After determining the resting motor threshold (RMT), we applied iTBS, at 80% RMT. We navigated to the individual left DLPFC target using an online neuronavigation system (Visor 1 software, ANT Neuro, Enschede, Netherlands). We obtained a pre-iTBS (baseline) rsfMRI scan (R0) followed by three post-iTBS rsfMRI scans (R1, R2, R3). Subjects completed the Positive and Negative Affect Schedule (PANAS^43^) both before and after the experiment on session 2 and session 3. This allowed us to follow any short-term changes in the subjects’ mood potentially influenced by iTBS. Figure 1 schematically shows the study design.

### rTMS protocol

We delivered iTBS using a MagVenture X100 with Mag-option and a “figure of 8” MCF-B65 cooled butterfly coil at the targets selected using each individual subject’s rsfMRI (see ref. ^19^). We used stimulation parameters from Li C-T et al.^5^ (3 pulses burst at 50 Hz delivered at 5 Hz for 2 s with an 8 s inter train interval, total 60 trains delivered during 9 min 30 s). For the sham condition, we rotated the coil by 180° along the handle axis as described elsewhere^19^. We did not employ the usual method of rotating the coil by 90°, as a complete 180° rotation allowed us to make the sham condition look as similar as possible to the real condition, for more effective blinding of subjects.

### Image acquisition

We collected functional data and, in between the rsfMRI scans, structural (T1- and T2-weighted scans with 1-mm isotropic resolution) data with a 3T MR scanner (Magnetom TIM TRIO, Siemens Healthcare, Erlangen, Germany) using a 32-channel head coil. The T2*-weighted multi-band gradient echo echo-planar imaging sequence provided by the Center for Magnetic Resonance Research of the University of Minnesota^44,45^ had the following parameters: repetition time of 2.5 s, echo time of 33 ms, flip angle of 70°, 60 axial slices with a multi-band factor of 3, 2 × 2 × 2 mm^3^, FOV of 210 mm, with 10% gap between slices and posterior to anterior phase encoding. The rsfMRI data were acquired with 125 volumes in approx. 5 min. The gradient echo field map was acquired with repetition time of 603 ms, echo times of 4.92 ms (TE 1) and 7.38 ms (TE 2), flip angle of 60°, 62 slices, FOV of 210 mm, 2 × 2 × 2 mm^3^, with 10% gap between slices and anterior to posterior phase encoding.

### Imaging data analysis

We preprocessed the rsfMRI data, using SPM12 (http://www.fil.ion.ucl.ac.uk/spm/software/spm12/) and MATLAB (The MathWorks, Inc., Natick, MA, USA), to execute the following state-of-the-art steps: slice time correction, motion correction, gradient echo field map unwarping, normalization, and regression of motion nuisance parameters, cerebrospinal fluid and white matter. Following this, we temporally concatenated the data for group independent component analysis (ICA) with FSL 5.0.7 software^46^. We visually identified the independent component (IC) that best resembled the DMN and another IC that best covered the left DLPFC (IC-DLPFC). We back reconstructed this IC representing the DMN in the normalized rsfMRI data of individual subjects, r-to-z transformed and compared across the groups using a factorial design ANOVA (Real [R0, R1, R2, R3] versus Sham [R0, R1, R2, R3]).

### Extraction of parameter estimates (functional connectivity strengths)

**Fig. 2:**
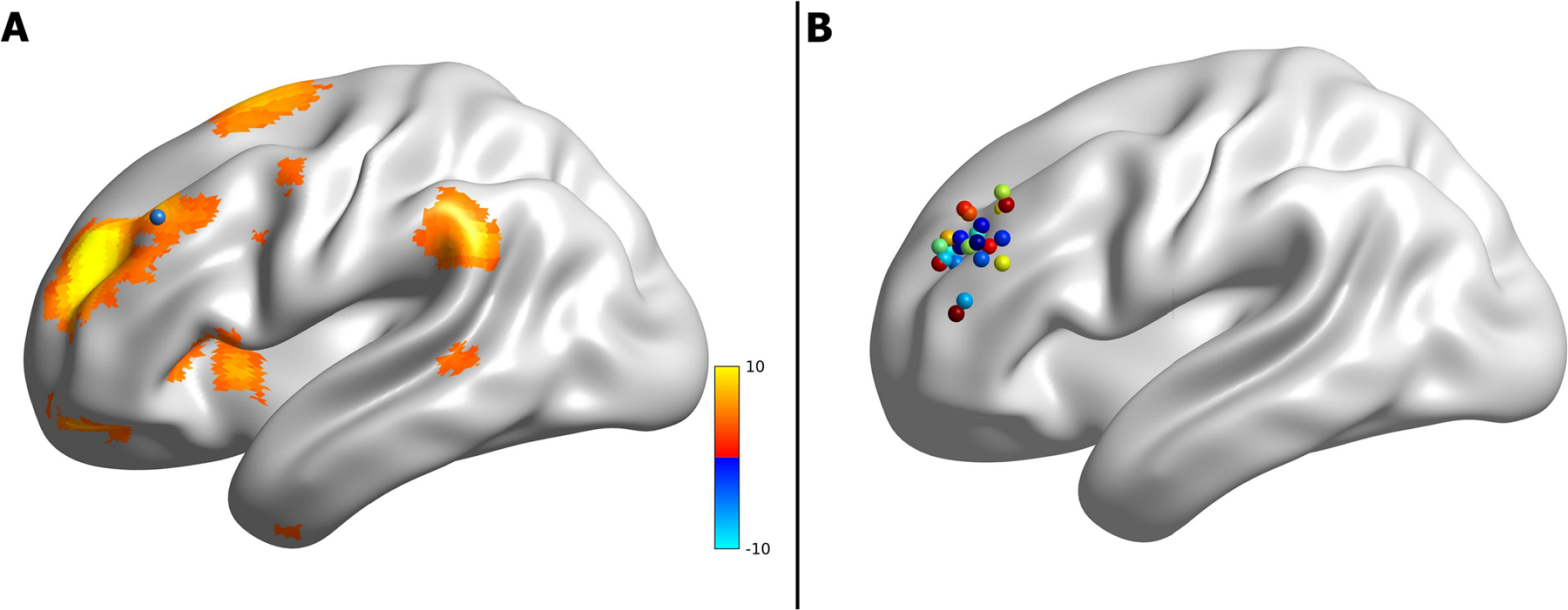
Personalized left DLPFC sites. **a** An example of IC-DLPFC (warm colors) for a single subject from which the parameter estimates of personalized stimulation site (blue sphere) were extracted. **b** Personalized left DLPFC stimulation sites of all subjects from which parameter estimates were extracted.

We used MarsBar^47^ to extract the parameter estimates (beta weights) of the rostral anterior cingulate cortex (rACC; 5 mm radius sphere) and subgenual anterior cingulate cortex (sgACC; 5 mm radius sphere) centered on independent coordinates from a meta-analysis of functional large-scale networks in depression^48^ and our previous work on 10 Hz rTMS effects on DMN^19^, respectively. The parameter estimates for the individual left DLPFC sites were extracted using 2 mm radius sphere region of interest (ROI) centered around the targets, in line with our previous work^19^. Figure 2a highlights an example subject showing the IC-DLPFC (in warm color), the network from which the parameter estimates using an individual left DLPFC target ROI (blue sphere) is extracted. Figure 2b shows all the ROIs that were used for parameter estimate extraction.

### Statistical analysis

Previous data on twenty-three subjects have shown adequate power to detect the differences in DMN after rTMS^19^. Using a factorial design ANOVA in SPM12 we compared the time windows of rsfMRI across real and sham conditions, and report results surviving a statistical threshold of *p* < 0.05 FWE whole-brain corrected for multiple testing. We ran Pearson’s correlation tests between rACC functional connectivity strengths and the HA domain of the TCI using MATLAB. We used R to run two-way t-tests to compare the scores from YMRS, HAM-D, MADRS, PANAS, VAS, and BDI II for real and sham stimulation sessions.

## Results

Twenty-nine healthy subjects (11 females, mean age of 28 ± 8 years) signed up for the study. Two subjects (both females) were dropped from the study due to failure to locate their personalized left DLPFC target and one subject (male) dropped out of study due to discomfort from stimulation. Thus, 26 subjects were included in final analysis, none of whom reported any adverse effects during or after stimulation.

### Functional connectivity changes after real stimulation

**Fig. 3:**
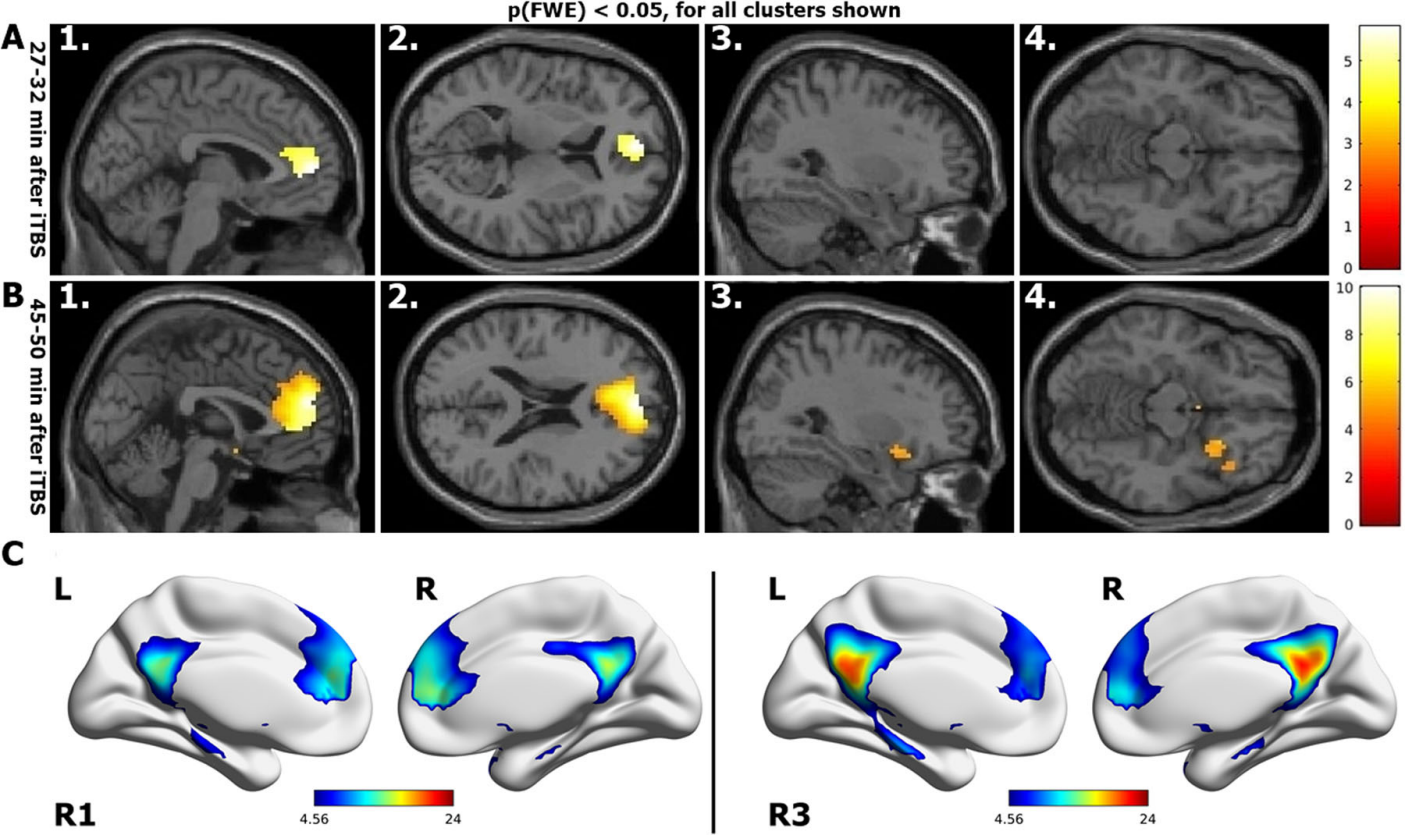
Functional connectivity results. Regions that show reduced functional connectivity to DMN after stimulation (real-sham condition, whole-brain corrected p_FWE_ < 0.05): (**a**1-2) About 27 min after iTBS, the rACC and dACC disengage from the DMN. (**b**1-2) About 45 min after stimulation, the functional connectivity has further reduced, extending to the mPFC and (**b**3-4) the right AI. **c** DMN during R1 rsfMRI and R3 rsfMRI session after real stimulation.

After a full single session of iTBS (1800 pulses) we observed reduced functional connectivity of the rACC and dorsal ACC (dACC) with the DMN, during the R2 rsfMRI session (27–32 min post-stimulation) when compared to R1 rsfMRI session (10–15 min post-stimulation) (Fig. 3 [A1-A2]). Even more interesting was the effect on the functional connectivity of DMN during the R3 rsfMRI (45–50 min post-stimulation), which increased in spatial extent. During R3, the area of significantly reduced functional connectivity of the DMN spread to include the medial prefrontal cortex (mPFC) and frontal poles, as seen in Fig. 3 [B1-B2]. Additionally, the right anterior insula (AI) showed decreased functional connectivity to the DMN during R3 rsfMRI (Fig. 3 [B3-B4]). These findings were not seen in the sham condition. Changes in clinical scales were neither expected nor identified. Also, it is important to note that when comparing the DMN only across real iTBS rsfMRI sessions without sham correction, we see the same regions decoupling from the DMN (Supplementary Fig. 1), except by smaller mPFC and larger right AI blobs in the R2 rsfMRI. In this case, the decoupling of the right AI is more pronounced, showing significantly reduced functional connectivity even during the R2 rsfMRI.

### Functional connectivity changes in the left DLPFC and the rACC along time

**Fig. 4:**
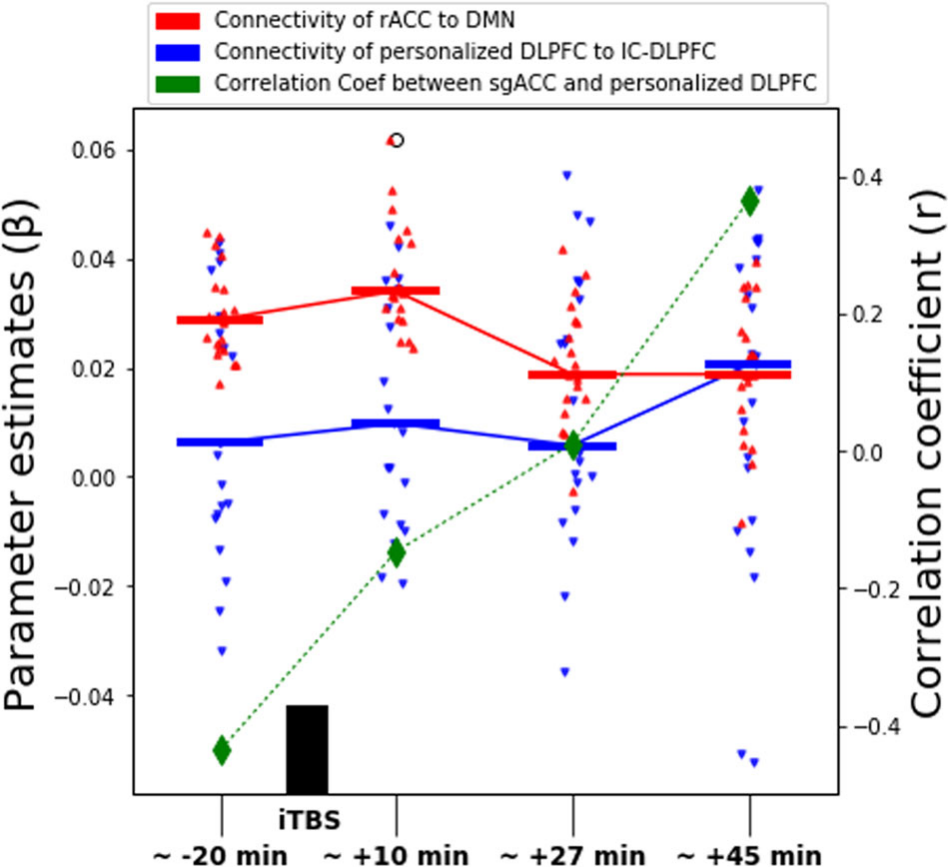
Parameter estimates of left DLPFC and rACC. Left axis shows the parameter estimates of left DLPFC (blue) and rACC (red) of IC-DLPFC and DMN, respectively. Dots represent the individual values and horizontal lines depict the median of the parameter estimates for the respective rsfMRI window. The right axis plots the correlation coefficients between the DLPFC and the rACC, showing that the effect of a single session of iTBS progressively changes the correlation between these ROIs from negative to positive.

To have a better understanding of the effects of iTBS, we extracted the parameter estimates of two ROIs in the real condition: the stimulated left DLPFC site and the rACC. We used a spherical ROI of 2 mm radius centered at the left DLPFC target to extract its parameter estimates from the IC-DLPFC (see methods for definition of IC-DLPFC). We extracted the parameter estimates of the rACC from the DMN. Following an earlier study of the ACC with a 10 Hz protocol^19^, a spherical 5 mm radius ROI was used with coordinates obtained from an independent meta-analysis^48^. The plot (Fig. 4) shows that the DMN functional connectivity of the rACC increases from the R0 to the R1 rsfMRI window. Subsequently, a functional connectivity decrease in the rACC from R1 to R2 is sustained until R3. A statistically insignificant increase in the IC-DLPFC functional connectivity of the left DLPFC from R0 to R1 is also seen. This functional connectivity returns to a value close to baseline during R2 and increases during R3 rsfMRI. The green dashed line represents the correlation coefficients between the parameter estimates of the sgACC and the left DLPFC. It shows that as the effect of iTBS becomes more prominent, the correlation between these regions goes from negative to more and more positive, not returning to baseline within 50 min after iTBS. We explored the changes in functional connectivity of these regions for sham condition (Supplementary Fig. 2) and observed minor changes in the median of parameter estimates (ranging between 0 and 0.02) and in the correlation coefficient (between 0 and 0.17). However, the functional connectivity fluctuates around the baseline during all rsfMRI sessions.

### Harm avoidance—a predictor of iTBS response?

**Fig. 5:**
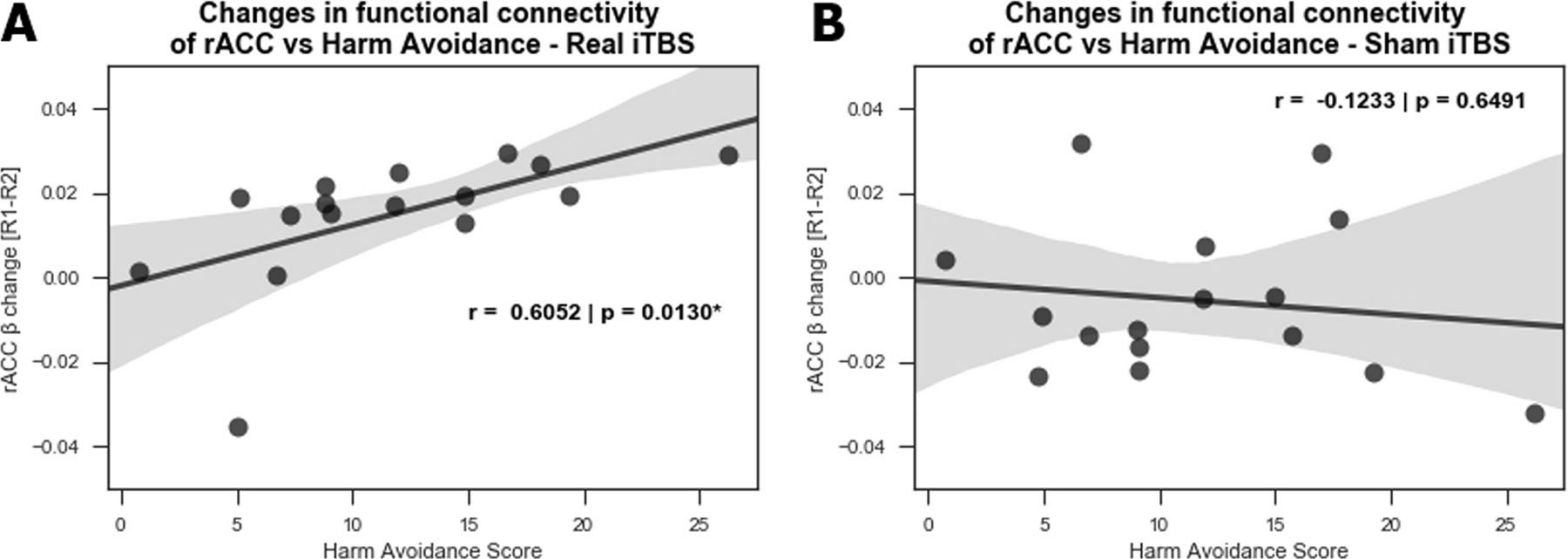
Correlation to harm avoidance scores. Correlation between the HA score and the changes observed in rACC functional connectivity during R2 rsfMRI compared to R1 rsfMRI for (**a**) real and (**b**) sham conditions. A significant positive correlation is observed after real stimulation only.

The mean and SD (11.625 ± 6.42) of the sample are representative of a healthy population^49,50^. A chi-square test for normality indicates that the HA is normally distributed (*p*-value = 0.962). Our previous work has identified a negative relationship between HA scores and the changes induced by 10 Hz rTMS in the right sgACC during R2 rsfMRI compared to R1 rsfMRI^19^. We hence explored if such a relationship existed also between the HA scores of subjects in the current study and the observed decrease in the functional connectivity of rACC during R2 rsfMRI compared to R1 rsfMRI. We identify a positive correlation between the HA measure and the decrease in functional connectivity of the rACC, only after real stimulation (*r* = 0.6052, *p* value = 0.013) but not after sham stimulation (*r* = −0.1233, *p* value = 0.6491). This indicates that the higher the HA score of the subjects the more they showed a decrease in their rACC functional connectivity to DMN (Fig. 5).

## Discussion

In this double-blind, sham-controlled study, we have determined for the first time the connectivity changes of the DMN in the healthy brain for up to 50 min after iTBS (1800 pulses protocol). As expected, after left DLPFC stimulation (Fig. 2b) we see a decrease in the functional connectivity of the DMN, mainly with the rACC and dACC during the R2 rsfMRI window (Fig. 3 A1-A2, about 27–32 min after stimulation). This decrease is sustained in the dACC and additionally extends to the mPFC and right AI during the R3 rsfMRI window (Fig. 3 B1-B4, about 45–50 min after stimulation). In agreement with the literature^18,51^, we see at baseline a negative correlation between the parameter estimates of sgACC and the left DLPFC (Fig. 4, green diamond at ~20 min before iTBS). As the functional connectivity changes in both left DLPFC and rACC within their own networks (Fig. 4, red and blue curves), the negative correlation between sgACC and left DLPFC becomes progressively positive (Fig. 4, green dashed curve). Finally, we observe a positive correlation between the HA score and the connectivity changes observed with the DMN in the rACC (Fig. 5a), which implies that this measure can possibly predict the magnitude of functional connectivity changes induced by iTBS in the rACC.

A dynamic system known as the triple network model has been suggested to explain the fast adaptive qualities of the brain^52,53^. According to the triple network model, a task positive network corresponding to the central-executive network (CEN) is active when the brain is engaged in cognitive tasks or allocating attention to external stimuli^22,54^. The DMN (aka task negative network) is active antagonistically to the task positive network, when resources are internally allocated during introspective thoughts or autobiographical memories^55^. A dynamic interplay between the task positive and task negative network is required to quickly reallocate resources towards internal or external stimuli according to immediate demands. It has been shown that a “circuit breaker” role is played by the salience network (SN)^53^, with the dACC and right AI as the main network nodes along with rACC involved with affective processing^33,56^. In this work we identified these regions as being decoupled from the DMN in healthy subjects after a single session of a prolonged iTBS protocol (1800 pulses) (Fig. 3). Although the changes evidenced here may not directly translate to the context of psychopathology, it was intriguing to see these nodes as part of our results. The AI is considered to be the essential hub of the SN because it mediates the information flow across the brain to different networks and switches between central-executive and DMNs^52,53,57^.

In depression, the SN shows aberrant functional connectivity to the DMN and CEN^22,31,58^. One of the network-based hypotheses of depression conjectures that the increased interaction between the SN and the DMN results in pathologically increased allocation of resources to negative information about the self, e.g. ruminative thoughts^27,59^. Considering the proven efficacy of iTBS for treatment of depression and speculating that the effects seen in healthy participants would extend to patients, the mechanism by which iTBS may initially influence the symptomatology of depression could be by “normalizing” the pathologically increased interaction between the SN and the DMN. In line with this reasoning, Iwabuchi et al.^60^ has shown in patients with depression that fronto-insular and SN connectivity interactions correlated positively with HAM-D score change at the end of a 4-week iTBS protocol. They have also described that better clinical outcomes are associated with reduced connectivity between dorsomedial prefrontal cortex (DMPFC) and bilateral insula^58^. Their results in conjunction with ours highlight the importance of investigating the AI and dACC as SN nodes involved in responsiveness to iTBS.

Using a different approach, Baeken et al.^61^ have shown that the functional connectivity of the sgACC and medial orbitofrontal cortex (mOFC) is increased during accelerated iTBS in depression patients. Their results stem from seed-based analysis of the sgACC after iTBS. One possible reason for the discrepancy between their and our results may be the method of analysis, as seed-based analysis focuses on the functional connectivity of a predefined ROI while ICA allows exploration of functional connectivity changes of the whole brain without having to pre-define a ROI. In contrast to our previous work that identified the sgACC as the main region decoupled from the DMN after a single session of personalized 10 Hz rTMS^19^, the strongest changes in connectivity after iTBS are not with the sgACC, but rather the rACC and dACC as mentioned above. However, due to the relevance of the sgACC, we further explored the beta weights from this region and evaluated its relation to the left DLPFC at baseline and up to 50-min after stimulation. We evidenced a shift of correlation between these regions from negative to positive within the observation time (Fig. 4, green dashed curve). This suggests the participation of the sgACC in the effects driven by iTBS, even though it is not directly engaged by it. The striking similarity between the red curves seen in the rACC after iTBS (Fig. 4) and in the sgACC after 10 Hz TMS (Fig. 5 in Singh et al.^19^) suggests that sgACC is rather the first target after 10 Hz rTMS (under standard dose of 3000 pulses). Another important aspect that might have contributed to differences between our and the results of Baeken et al.^61^ is that we stimulated functionally relevant sites within the left DLPFC, as opposed to their structural selection of stimulation sites. Of course, the most profound difference is that our study closely evaluated connectivity changes after one session of iTBS in healthy subjects, whereas Baeken et al.^61^ evaluated patients with depression after 20 stimulation sessions. It must be considered that the complexities associated with the underlying pathophysiology of depression could have contributed to differences in how iTBS interacts with brain regions and networks. Our results shed light on other relevant regions that respond to a single session of iTBS in the healthy brain. Future work examining brain networks in patients before and after 20 iTBS treatment sessions would likely close these knowledge gaps.

By rounding up results from our previous work^19^ and current study, 10 Hz rTMS disengages the anterior nodes of DMN and thus affects sgACC and DMN connectivity. 10 Hz rTMS could hence reduce sadness, rumination and self-directed thought processes. In fact, we have seen a trend of reduced negative affect after 10 Hz rTMS in healthy subjects. ITBS, however, acts via a different brain network. It does not disengage DMN with itself directly but reduces the communication between nodes of SN and DMN. Therefore, we assume that after iTBS subjects would engage less in affective content of external information due to reduced communication between SN and DMN. However, this needs more precise testing in future studies.

We also evidenced a positive correlation between the HA score on the TCI and changes in the functional connectivity of the rACC and the DMN (Fig. 5a). This indicates that the higher the subjects scored on the HA domain, the stronger the reduction in observed functional connectivity. This correlation indicates that it might be possible to utilize HA to predict the extent of DMN-rACC coupling changes induced by iTBS. Interestingly, the correlation between connectivity changes and HA scores replicates the time window in which this was seen in an independent sample using 10 Hz rTMS^19^, although in opposite direction and in a different brain region, the sgACC.

The opposing results likely stem from the fact that 10 Hz rTMS and iTBS involve different brain networks in their action as discussed above. Previous works have shown positive relation between HA and rumination^62,63^, higher levels of which are associated with lower intra anterior DMN functional connectivity^64^. Huggins et al.^65^ have shown that HA correlates with the strength of anticorrelation between an SN node and DMN. This implies healthy subjects with higher HA would have lower anterior DMN functional connectivity and higher SN-DMN connectivity. Consequently, subjects with higher HA would presumably have higher rumination and greater propensity for negative valence stimuli, while subjects with lower HA would display the opposite. In this line, higher anterior DMN connectivity related to lower HA would respond more pronouncedly to 10 Hz rTMS as it directly disengages the functional connectivity between sgACC and DMN^19^. Conversely, higher SN-DMN connectivity related to higher HA would respond more conspicuously to iTBS as it disengages the nodes of SN from DMN. Following this reasoning, we propose that high HA subjects would respond oppositely to 10 Hz rTMS and iTBS.

Thus, healthy subjects with higher rumination, and anxiety and vigilance towards external stimuli (high HA) are more likely to respond to iTBS via disengagement of SN nodes from DMN. While those with lower disposition for rumination, anxiety, and vigilance towards external stimuli (low HA) are more likely to respond to 10 Hz rTMS, which directly disengages higher functional connectivity between sgACC and DMN. Considering the opposite relationship between HA and DMN effects from 10 Hz rTMS and iTBS, we speculate that HA scores may facilitate identification of subjects who will present stronger DMN changes in response to rTMS protocols. However, given clinically depressed population have a higher HA scores^38^, an elevated DMN connectivity^15^, and increased rumination^27^ and self-directed thoughts, such predictive use of HA requires rigorous testing. We speculate that patients with depression having HA greater than the depressed population’s average might more favorably respond to iTBS while those with HA lower than the depressed population’s average would respond better to 10 Hz rTMS. If such results hold true for clinical population receiving multiple sessions of rTMS, then HA could be used to determine beforehand who would benefit most from one stimulation protocol or the other. We hope future research in precision medicine will investigate this aspect, considering the direct clinical application and potential relevance to improving treatment response.

There are limitations to our study. For ethical reasons we applied a single session of 1800 pulses iTBS for uncovering its effect on the DMN in healthy brains, since applying 20 sessions of iTBS as is done in patients^66^ would not be prudent. Also, the fact that the non-stimulation side of an MCF-B65 coil can have non-zero current implies that sham condition was not completely passive. It is however unlikely that sham condition has biased our main results, considering that (a) connectivity changes from sham stimulation do not show any changes analogous to effects from real condition; and (b) the same specific nodes display connectivity changes after real stimulation without sham comparison (Supplementary Fig. 1). The diseased state of the brain, e.g. in depressive state, is also likely to influence interactions between brain networks in response to multiple sessions of iTBS. Therefore, assumptions based on healthy samples must be made cautiously. Finally, we did not expect to observe any significant neural effects from iTBS beyond 50 min after stimulation, however our results indicate that iTBS effects are strongest in the rsfMRI scan from 45 to 50 min after stimulation. This information points towards the fact that iTBS effects likely last beyond the time window of our study and should be further examined in future studies.

In conclusion, by means of a double-blind, sham-controlled crossover study involving healthy subjects, we show that a single session of iTBS results in decoupling of the rostral/dorsal ACC, followed by the mPFC and the right AI, with the DMN. The interaction between the sites of stimulation at the left DLPFC and the sgACC shows a progressive shift from negative to positive correlation. Lastly, connectivity changes in the rACC induced by a single real session of iTBS in the healthy brain positively correlated with the HA score on the TCI scale.

## Supplementary information

Supplementary Material

## Data Availability

Owing to restrictions in the data sharing consent obtained from the participants of the study, the datasets generated and analyzed cannot be made publicly available.
