# Supplementary material for: Default mode network alterations after intermittent theta burst stimulation in healthy subjects
Source: Transl Psychiatry. 2020 Feb 24;10:75. doi: 10.1038/s41398-020-0754-5 (PMC7040002; doi:10.1038/s41398-020-0754-5)
Supplement: Supplementary file 1 — Supplementary Material [file 41398_2020_754_MOESM1_ESM.docx]

**Title: Default mode network alterations after intermittent theta burst stimulation in healthy subjects**

**Authors:** Aditya Singh^1^, Tracy Erwin-Grabner^1^, Grant Sutcliffe^1^, Walter Paulus^2^, Peter Dechent^3^, Andrea Antal^2^, Roberto Goya-Maldonado^1,^*

**Affiliations:**

^1^Laboratory of Systems Neuroscience and Imaging in Psychiatry, Department of Psychiatry and Psychotherapy of the University Medical Center Göttingen.

^2^Department of Clinical Neurophysiology of the University Medical Center Göttingen.

^3^Core facility ‘MR-Research in Neurology and Psychiatry’, Department of Cognitive Neurology of the University Medical Center Göttingen.

*To whom correspondence should be addressed:

*Dr. Roberto Goya-Maldonado*

*Laboratory of Systems Neuroscience and Imaging in Psychiatry (SNIPLab)*

*Department of Psychiatry and Psychotherapy*

*University Medical Center of Göttingen*

*Von Siebold Straße 5, 37075, Göttingen, Germany*

*Tel: +49 (0) 551-39-22244*

*(*[*roberto.goya@med.uni-goettingen.de*](mailto:roberto.goya@med.uni-goettingen.de)*)*


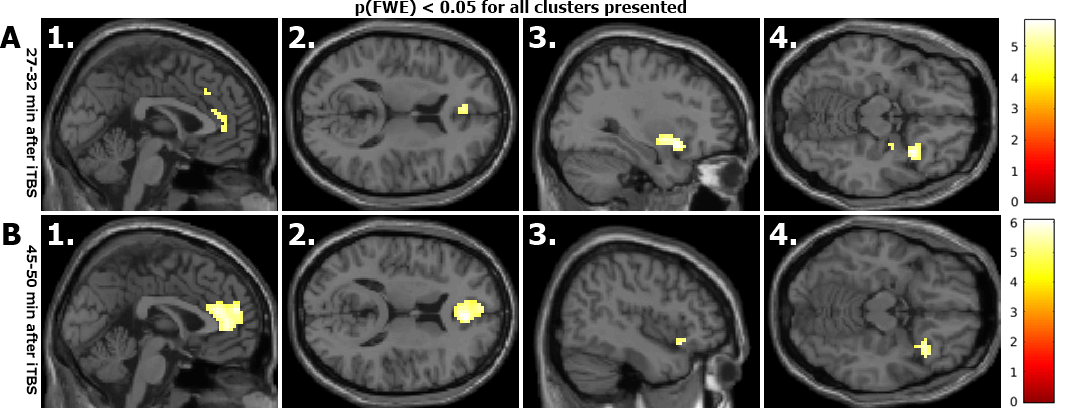
**Supplementary Materials**

Supplementary Figure 1: Changes in functional connectivity of default mode network (DMN) after real iTBS without comparison to sham condition. The effects of real iTBS (without sham comparison) are very similar to those obtained when real iTBS is compared against sham iTBS (Figure 3), except by smaller mPFC and larger rAI blobs in the R2 rsfMRI (Suppl. Figure 1 – A1-A4).


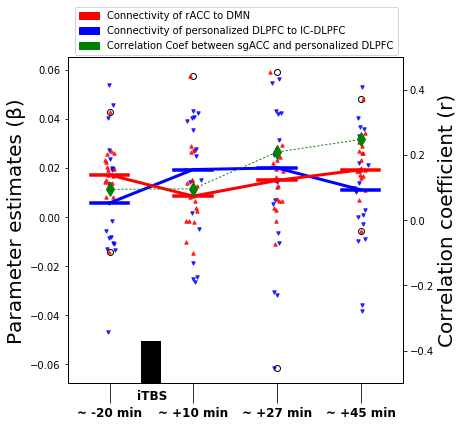


Supplementary Figure 2: Left axis shows the parameter estimates of left DLPFC (blue) and rACC (red) of IC-DLPFC and DMN, respectively, in the sham condition. Dots represent the individuals and horizontal lines depict the median of the parameter estimates for the respective rsfMRI window. Only minor changes in the median of parameter estimates ranging between (0-0.02) and correlation coefficient between (0-0.17) are observed. This implies the functional connectivity fluctuates around the baseline during all rsfMRI sessions and the interaction between sgACC and personalized left DLPFC is also not affected by sham iTBS.
